# Recurrent acute pancreatitis secondary to sesame allergy

**DOI:** 10.1097/PG9.0000000000000042

**Published:** 2021-01-13

**Authors:** Mohammed Alamrani, Mona Alasmi, Abdullah Alangari

**Affiliations:** From the * Division of Gastroenterology, Department of Pediatrics, College of Medicine, King Saud University, Riyadh, Saudi Arabia; † Division of Allergy and Immunology, Department of Pediatrics, College of Medicine, King Saud University, Riyadh, Saudi Arabia.

Food allergy is a common health problem affecting up to 5% of population in Europe and the United States. It usually involves multiple systems most commonly the skin (urticaria and angioedema), then the respiratory (cough and shortness of breath), cardiovascular (tachycardia and hypotension), and gastrointestinal system (vomiting, diarrhea, and abdominal pain) (1). The most common food allergens are cow milk, hen egg, wheat, fish, soy, peanuts, tree nuts, and shellfish (2). Acute pancreatitis is an extremely rare manifestation of food allergy. It was first reported in dogs in 1933 (3). Following this, a few sporadic cases were reported in humans caused by allergies to milk, banana, kiwi, eggs, shrimp, peanuts, and wheat (4). So far, only 17 cases of food-induced pancreatitis were reported in the literature (4).

## CASE REPORT

**FIGURE 1.**
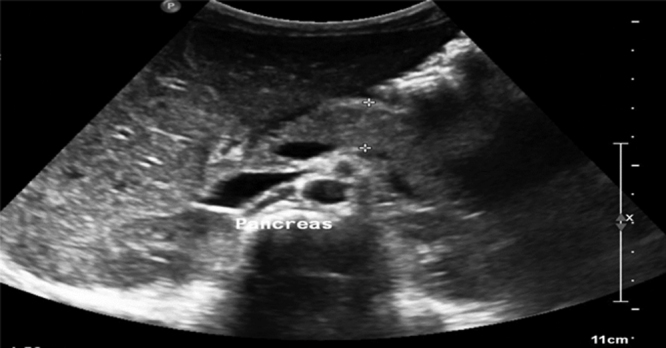
The pancreas appears bulky in size without focal lesions and no pancreatic fluid seen.

Our patient is a 6-year-old boy who is known to have asthma and allergy to milk, eggs, bananas, and peanuts. His symptoms included mild skin rash, itching, and irritability. His family history was unremarkable, except for the presence of banana allergy in his father. His asthma treatment included albuterol and fluticasone inhalers. Our patient had 7 visits to the emergency department due to abdominal pain during 2016–2017 and was hospitalized after every visit. All the episodes were similar, involving acute severe epigastric abdominal pain associated with vomiting without clear aggravating or relieving factors. There was no history of diarrhea, fever, skin rash, edema, respiratory or cardiovascular related symptoms, or trauma. His vital signs were stable. Abdominal examination revealed epigastric tenderness. Examination of other systems was unremarkable. During each episode, his pancreatic enzymes were very high. Other laboratory parameters were within normal (Table 1). Each episode was treated within 24–48 hours of onset by keeping the patient nil per oral for few hours, while providing intravenous fluids, and analgesics. During the third episode, abdominal ultrasonography revealed bulky pancreas without focal lesions (Fig. 1). Common bile duct caliber was normal. Few days later, magnetic resonance cholangiopancreatography was normal. Genetic testing of known inherited causes of chronic pancreatitis (*PRSS1*, *SPINK1*, and *CFTR*) was normal. The patient was eventually diagnosed with idiopathic recurrent pancreatitis. However, during the seventh episode, sesame ingestion was linked to the patient’s symptoms for the first time when his mother recalled that his symptoms of acute abdominal pain started within 1 hour after he consumed sesame cookies, and it was the only food that he consumed in the preceding few hours. She also recalled, in retrospect, that he consumed sesame shortly before the third episode in addition to other food items, but could not recall his food consumption before the remaining episodes. Sesame-specific IgE by the radioallergosorbent test was 86.3 kU_A_/L (normal <0.35 kU_A_/L), his total IgE level was also high (616 kU/L), and his skin prick test to sesame commercial extract showed a wheal diameter of 3 mm (normal saline negative control is 0. and, histamine positive control is 6 mm). In October 2017, the patient’s family was advised that he should avoid sesame. Since then, the patient has had no more episodes of acute pancreatitis. An oral challenge test was not performed because of family refusal. He was finally diagnosed with sesame-induced acute recurrent pancreatitis.

**Table 1.** Laboratory Tests During Each Episodes

Parameter	First Episode	Second Episode	Third Episode	Fourth Episode	Fifth Episode	Sixth Episode	Seventh Episode	Reference Range
Amylase	484	315	1768	650	650	1433	1948	25–125 U/L
Lipase	2680	1468	7840	9851	7749	8222	10398	73–393 U/L
AST	116	132	103	125	ND	ND	109	20–65 U/L
ALT	33	28	18	26	25	25	25	12–37 U/L
Total bilirubin	9	5	3.2	4.95	5.37	5.93	5.5	3–17 μmol/L
Direct bilirubin	3	1	0.87	1.28	1.67	1.68	1.4	0–5 μmol/L
GGT	18	19	20	21	18	13	17	15–85 U/L
Total protein	62	70	65	70	68	66	69	60–80 g/L
Albumin	36	41	36	37	41	35	34	30–50 g/L

ALT = alanine aminotransferase; AST = aspartate aminotransferase; GGT = gamma-glutamyl transferase; ND = not done.

## DISCUSSION

Our patient’s diagnosis was based on careful medical history of food ingestion few hours before the pancreatitis episodes, supportive laboratory and skin testing findings, and cessation of pancreatitis episodes after withdrawal of sesame. This is the first case of sesame-induced acute pancreatitis in the literature. The diagnosis of food-induced pancreatitis could be challenging because it is extremely rare and needs a high index of suspicion. It can even be more challenging when there are no systemic manifestations beside the gastrointestinal system such as skin, respiratory, or cardiovascular symptoms as in our case. Quick improvement has been noted in most reported cases after keeping the patients nil per oral and on intravenous fluids (5). The mechanism of food-induced pancreatitis remains unclear. One possible explanation was proposed by Inamura et al (6) that there is obstruction of the ampulla of Vater secondary to inflammation-induced swelling which causes bile reflux into the pancreatic duct. In their case, they performed esophagogastroduodenoscopy during an acute episode when they noted inflammatory changes in the ampulla of Vater and biopsy from the site showed a high number of mast cells infiltrate (6). Most patients stopped from having new episodes of pancreatitis after avoiding the culprit food (5). Other possible supportive treatment options include the use of corticosteroids and mast cell stabilizers; however, these medications resulted in only temporary relief of symptoms (5). In conclusion, food-induced pancreatitis should be considered in any patient with idiopathic recurrent pancreatitis and food allergies. More research is needed to further understand this rare entity.

## ACKNOWLEDGMENT

We would like to thank the Deanship of Scientific Research, King Saud University for their support through the research group project No. RGP-190.
